# HIV care provider perceptions and approaches to managing unhealthy alcohol use in primary HIV care settings: a qualitative study

**DOI:** 10.1186/s13722-019-0150-8

**Published:** 2019-06-07

**Authors:** Natalie E. Chichetto, Zachary L. Mannes, Megan K. Allen, Robert L. Cook, Nicole Ennis

**Affiliations:** 1 Department of Medicine, Vanderbilt University Medical Center, 2525 West End Avenue, Suite 450, Nashville, TN 37203 USA; 2 Department of Clinical and Health Psychology, College of Public Health and Health Professions, University of Florida, Gainesville, FL USA; 3 Department of Epidemiology, Colleges of Public Health and Health Professions and Medicine, University of Florida, Gainesville, FL USA; 4 Department of Behavioral Sciences and Social Medicine, Florida State University College of Medicine, Center for Translational Behavioral Science, Tallahassee, FL USA

**Keywords:** Addiction treatment, Alcohol, Alcohol screening, Education, HIV, Knowledge

## Abstract

**Background:**

HIV care providers often serve as the specialist and the primary care point-of-contact for persons living with HIV (PLWH) and unhealthy alcohol use. The purpose of the present qualitative study was to understand HIV care provider perceptions and approaches to managing unhealthy alcohol use in HIV primary care settings.

**Methods:**

Using a semi-structured interview guide, in-depth interviews were conducted among 14 HIV care providers (5 medical doctors, 5 nurse practitioners/physician assistants, 2 medical assistants, 2 clinical administrative staff) in private and public HIV clinics, across urban and rural areas of Florida. Interviews were coded using a grounded theory approach with inter-rater consensus.

**Results:**

Six themes were identified. In summary, providers reported (1) inconsistent assessment of alcohol consumption, as well as (2) varying levels of confidence in self-report of alcohol use which may be affected by patient provider rapport and trust. While providers (3) acknowledge potential negative impacts of alcohol use on health outcomes and HIV treatment, providers reported (4) inconsistent recommendations regarding alcohol use among their patients. Lastly, providers reported (5) limited resources for patients with unhealthy alcohol use and (6) low confidence in their ability to help patients reduce use.

**Conclusions:**

Results from our study suggest salient differences in provider approaches to the assessment and management of unhealthy alcohol use in HIV primary care settings. Implementation of care for unhealthy alcohol use in these settings may be facilitated through use of clinically useful, validated alcohol use assessments and use of evidence-based recommendations of alcohol use/non-use among PLWH. Training in brief intervention techniques for alcohol reduction may increase provider confidence and support in the management of unhealthy alcohol use among PLWH.

## Introduction

Among persons living with HIV (PLWH), unhealthy alcohol use, ranging from at-risk use [> 14 (7) drinks per week for men (women)], binge drinking [> 4 (3) standard drinks on one occasion in men (women)] to alcohol use disorder (AUD) [1], is associated with lower adherence to antiretroviral therapy (ART), compared to abstinence [2]. Approximately 10–30% of PLWH receiving services at HIV primary care clinics report unhealthy alcohol use [3, 4] and up to 19% meet criteria for AUD [5]. Any alcohol consumption is inversely associated with engagement in HIV-related care at each step of the HIV care continuum [6, 7]. Further, unhealthy alcohol use is associated with accelerated disease progression by dampening the effectiveness of ART [8], resulting in poor treatment outcomes [9], including increased viral load [10] and poor immune functioning [10–15]. Aside from effects on HIV care and disease progression, unhealthy alcohol use is associated with greater risk for cardiovascular disease [16–18], non-AIDS defining cancers [19], neurologic disorders [20–22], and reduced life expectancy [23–27].

Despite accumulating evidence suggesting that unhealthy alcohol use has detrimental medical and psychosocial effects among PLWH, the management of this behavior remains a significant challenge. HIV care practitioners often serve as both the HIV specialist and the primary care provider for PLWH living with comorbid substance use, mental health, and chronic illness [28]. In fact, up to 84% of PLWH report that they prefer to receive such integrated care from their HIV care provider, as opposed to having a separate primary care doctor [28]. However, knowledge regarding effective assessment of and intervention for unhealthy alcohol use among HIV-care providers remains a concern. Although HIV care providers report an understanding of the links between alcohol use and HIV transmission (93%), as well as the ability to assess unhealthy alcohol use (85%), providers are unlikely to have received any formal training on how to manage unhealthy alcohol use (41%) and only 54% report an understanding of current recommendations regarding unhealthy alcohol use [29].

While there is great potential to address unhealthy alcohol use in HIV primary care settings, providers often miss unhealthy alcohol use among PLWH, especially in patients whose HIV is well-managed [30]. Only 10% of providers report using a formal screening tool to assess alcohol use [31]. By extension, AUDs are rarely treated in HIV primary care settings, with over two-thirds of providers never/rarely treating AUD and 70% referring patients to specialized treatment [31]. Further, a recent study of over 800,000 patients found that among those with unhealthy alcohol use, PLWH were 15% (RR 0.85, p < .001) and 37% (RR 0.63, p < .001) less likely to receive any evidence-based brief intervention or supportive medicine, respectively, compared to uninfected patients, controlling for confounding factors such as care utilization [32].

General primary care stakeholders, including patients and providers alike, agree that universal screening of substance use, including alcohol, is important to health care management [33]. However, existing qualitative research reveals significant barriers to screening and treatment of unhealthy alcohol use in primary care, including limited resources (access to treatment centers or specialty care) [33–36], lack of knowledge/experience or training [31, 33–35, 37, 38], attitudes regarding the role of primary care versus specialty care [34, 38], and alcohol use stigma felt by patients [33, 37, 38]. In contrast, facilitators for screening and treatment of unhealthy alcohol use include having tools and support for providers (i.e., behavioral staff, tools to enhance patient motivation) [38, 39], screening/treatment goals that are consistent with organization values and/or existing practices [36, 39], increased provider self-efficacy [38, 39], and specialty care accessibility [36].

Given the general lack of literature regarding treatment for unhealthy alcohol use in HIV care settings, and the high prevalence of unhealthy alcohol use and disparate treatment by HIV status, we sought to gain information on HIV care provider perceptions and approaches to managing unhealthy alcohol use in their HIV primary care clinic.

## Methods

### Participants

Participants were HIV care providers (those providing direct service or facilitation of HIV care services) recruited from private and public HIV primary care settings, in urban and rural areas across the state of Florida between January-May 2016, namely the Florida Departments of Health in Alachua (Gainesville) and Hillsborough (Tampa) Counties, as well as from Jackson Memorial Hospital (Miami) and University of Florida Health Shands Hospital (Gainesville). In order to gain an understanding of the collective perspective on care for unhealthy alcohol use in HIV primary care settings, we sought a diverse group of HIV care providers within all levels of the HIV care cascade, including medical doctors, registered nurse practitioners, physician assistants, medical assistants and clinic administrative staff who have worked with PLWH for at least 1 year. Further, the diversity of care providers serves as a data triangulation method, for which to assess validation of data. A combination of convenience and snowball sampling was used to recruit providers–providers affiliated with the Southern HIV and Alcohol Research Consortium (SHARC), a center located within the University of Florida Department of Epidemiology examining determinants of health outcomes of PLWH within the state for Florida, were contacted with information about the study and asked to suggest alternative providers who may be interested in participating. Initially, the healthcare providers were contacted and given information about the current project, including a Letter to Providers and additional flyers to provide to HIV care staff. If a provider was interested in participating, an interview was scheduled. Upon providing written informed consent on the day of the interview, participants were asked to complete a short demographic questionnaire and the interview started thereafter. While a target sample size of 12–16 was initially proposed, recruitment continued until saturation of themes [40] was reached among the providers in aggregate—at 14 participants.

### Procedure

Face-to-face, semi-structured interviews with HIV care providers were conducted for approximately 30–45 min. Participants were provided a lunch gift card (value $15) for their time. An interview guide was used to standardize all interviews, utilizing open-ended questions with prompts in order to elicit a discussion around provider perceptions and care practices related to alcohol consumption (Table 1). While a guide was used, the interviews were semi-structured, allowing for further elicitation of emerging themes and/or clarification of existing themes. Interviews were conducted by the lead and senior authors and were digitally recorded and professionally transcribed by an external transcription service. Transcriptions were spot checked for accuracy; an error rate of greater than 5% prompted re-transcription of interviews. No transcription exceeded this error rate threshold. All audio recordings were stored on a password-protected server and deleted after transcription. All information was de-identified, with a participant ID replacing the participant’s name on all study documentation. The study was approved by the University of Florida Institutional Review Board. Participants provided informed consent prior to participation.

**Table 1:** Interview script for HIV care provider perceptions

Alcohol use assessment	1. How often do you assess alcohol use in routine care?
	2. What questions do you ask?
Addressing alcohol use issues	3. In your opinion, how should we address alcohol use issues in the primary care setting?
	4. Who would be the key agents in addressing alcohol use issues?
Alcohol use and HIV-related outcomes	5. What have you observed regarding HIV outcomes among patients that use alcohol?
	6. What recommendations do you give to your patients regarding alcohol consumption?
	7. What kind of information are your recommendations based on?
	8. Would knowing how much your patients used alcohol affect your course of treatment for HIV management?
Alcohol use resources and treatment options	9. What resources or treatment options do you have in order to address heavy alcohol use?
	10. What resources or treatment options do you need in order to address heavy alcohol use?
	11. How often do (or would) you use such resources?

### Data analysis

A qualitative data management and analysis program, NVivo 10™ (QSR International Pty. Ltd., 2012) was used for all management and analysis of qualitative data. While there is a vast qualitative literature regarding alcohol-related care in general primary care [33–38], there is little literature regarding alcohol-related care in HIV care settings [31, 39]. Because of this, a Grounded Theory approach [40, 41] was utilized– as previous findings in the general primary care literature may not be generalizable to specialty HIV care settings. Initially, one investigator conducted line-by-line analysis to identify initial codes. After initial coding, two additional multidisciplinary (i.e., medicine and public health, epidemiology, and clinical and health psychology) investigators not present at the interviews or previously involved in the project coded and identified themes; all members subsequently discussed the codes and proposed themes, as well as resolved any differences in coding. Codes were then collapsed into final themes presented in this article.

## Results

### Participant demographics

A total of 14 providers were recruited from private and public primary HIV care clinics, in urban and rural settings across Florida. The sample was 36% (n = 5) non-Hispanic white, 43% (n = 6) Hispanic, and 21% (n = 3) Other race/ethnicity; 28% (n = 4) were aged 25–34 years, 43% (n = 6) 35–44 years, and 28% (n = 4) 45 + years. Providers included medical doctors (MD, n = 5), nurse practitioners (NP)/physician assistants (PA, n = 5), medical assistants (MA, n = 2), and clinical administrative staff (CA, n = 2). Of the sample, an even 50% were recruited from private versus public clinic settings, as well as in rural versus urban areas. Providers had worked in HIV primary care for a mean of 12.2 years (Table 2, median 14, range 1–28 years). On average, providers reported that 51.3% and 42.8% of their patient population experienced issues with mental health and substance use, respectively. On a scale of 0–100% confidence, the providers reported an average of feeling 60% confident that they could help their patients with unhealthy alcohol use.

**Table 2:** Sample demographic information

*Years of work experience as an HIV care provider*	
Mean	12.2
Median	14
Distribution	1–28
*Percentage of patients that experience a mental health issue*	
Mean	51.3%
Median	45%
Distribution	15–99%
*Percentage of patients that experience a substance use issue*	
Mean	42.8%
Median	37.5%
Distribution	10–95%
*How confident do you feel helping patients with alcohol use problems?*	
Mean	60%
Median	50%
Distribution	10–100%

### Emergent themes

(1) *Providers reported inconsistent assessment of alcohol consumption* Participants were asked how and when alcohol use was assessed in routine care. In terms of frequency of assessments, responses ranged from no assessment to assessment at every visit. Many providers (N = 6; NPs and MDs) reported assessing alcohol consumption periodically, but not at every visit. Two of these providers reported asking patients about alcohol consumption at their first appointment:

Provider 1: “So – if it’s a new patient or even a patient I’m inheriting, there are a few things I always touch on. One of them is if they are abusing any type of drug, whether it is IV [intravenous] or alcohol related. Always ask about that in terms of the past.”

Outside of the first visit, providers reported asking about alcohol consumption if the provider suspected an alcohol use problem:

Provider 1: [Ask about alcohol consumption] “if we suspect something… if it seems like they’re having a hard time.”

Provider 2: “Certainly we don’t [ask] at every each visit. I think we only do them if it’s a real problem. If someone comes in and says, ‘Hey, I have a problem’.

One provider reported an existing protocol that wasn’t always followed:

Provider 2: “We’re supposed to be doing that every year. I don’t think we’ve actually done it every year for the alcohol.”

Other providers reported that limited time prevented them from conducting an alcohol use assessment:

Provider 3: “Sometimes I just don’t have time for it [to ask about alcohol consumption].”

Few providers reported assessing alcohol use at each visit (n = 3; PA, NP, MD).

Provider 4: “Well we are always aware of it. It’s one of our – you know, topics that we include in every regular visit with the patient.”

Of the providers that reported assessing alcohol use (n = 9), most reported asking questions to ascertain the frequency of alcohol use and the quantity (n = 6):

Provider 3: “I ask ‘em, ‘Do you drink any alcoholic beverages? That includes beer, wine, wine coolers or other actual liquors.’ I ask them on average, how many a week. If it seems like they’re having a hard time, I say, ‘Do you drink every day, do you drink every other day?’ I try to get a good quantification. I ask ‘em about how many beverages and how many ounces those are.”

The other providers reported asking similar questions, in addition to assessing the type of alcohol that is consumed.

Provider 1: “I’ll ask how much do they drink on a daily basis – you know, are they drinking beer and wine versus hard liquor. Do they binge?”

Provider 5: “We ask the amount, for how long they have been drinking, for how many years, if they are trying to quit, what happened – what made them restart [drinking alcohol]?”

(2) *There were inconsistent perceptions of self-report accuracy which may be affected by patient–provider rapport and trust* When asked about alcohol assessment, several providers (n = 7; PA, NP, MD) indicated that an important barrier to assessment and use of information gained was accuracy of self-report. One provider expressed concerns of under-reporting:

Provider 6: “The alcohol use itself is under reported. I think for alcohol [self-report] is very – I think patients – they downplay a lot when they give you history of alcohol use.”

Other providers recognized that self-report may not be accurate, but choose to trust in what patients are reporting:

Provider 7: “– and one of the things I do with my patients when I ask them…they tell me ‘Okay, I’m not going to lie to you.’ And I said ‘Listen – no, no, no, you can lie to me. You lie to me, I won’t be able to help you but I have to trust you. I have to believe whatever you tell me. I believe you.’ – Because I think we have to trust each other, and that’s why they come to see me because they trust me, and I will reciprocate that”

One provider expressed that understanding the patients’ jargon was valuable in establishing this trust:

Provider 8: “– it helps in knowing what’s the latest drug, drink – you know – because if you speak the lingo then they meet you halfway and they don’t see that you’re judging them – you know, you’re just having a conversation.”

Other providers felt that their patients were open and honest about their drug and alcohol use:

Provider 9: “All [of] my patients are upright, they will say ‘I used yesterday, or I used last night.’ They also know I ask and I expect them to tell me the truth and they do. They are so honest with me, and… I tell them ‘Be honest with me, I don’t care that you use, it doesn’t faze me’.”

(3) *Providers acknowledge potential negative impacts of alcohol use on health outcomes and how HIV-infection is treated* Providers were asked to describe what they had observed regarding HIV outcomes among patients that used alcohol. Providers were also asked how knowing their patient’s used alcohol would affect treatment and HIV management. Many providers (n = 9; CA, PA, NP, MD) reported that alcohol consumption among their patient population influenced medical compliance, mainly through accidentally or intentionally missing their ART dose or missing appointments:

Provider 1: “It’s the patients that have serious alcohol abuse issues; it begins to be a problem because they forget to take their medication or they’re just out of it and on a binder for a few days and they don’t take their medications.”

Also:

Provider 10: “Sometimes patients feel like, [I’m going drinking today, so I maybe be pro-active and I’m not going to take my medication]. Cause they feel, [the medication can hurt my liver, drinking is going to hurt my liver, but I’m going to drink, so I just won’t take my medication today]. I think that even my moderate drinkers sometimes are irresponsible as far as their care. They may miss appointments.”

Provider 11: “When they [patient] are in care and then all of a sudden they just drop out of care again, we know what’s going on [regarding alcohol use].”

Providers (n = 4; PA, NP, MD) mentioned noticeable differences in viral load suppression among their patients that drink:

Provider 3: “I can think of one particular example. This guy drinks all of the time. He comes to clinic every time smelling like beer. Every – every time. He comes to clinic almost difficult to understand. I’m like, Oh my God! Did you drive yourself? His viral loads are detectable.”

Also:

Provider 1: “They are the patients I worry about because they have detectable viral loads and develop resistance and it just becomes a really bad cycle.”

Several providers (n = 6; PA, NP, MD) mentioned the need to consider altering their patients’ ART medication regimen due to heavy drinking or problems caused by alcohol use (i.e. ART resistance due to medical non-compliance):

Provider 8: “I mean, when you have non-compliance you start dealing with resistance and your options for treating them become less and less. But, you would want to try and simplify the regimen as much as possible to decrease their burden – you know – to try and again, meet them in the middle. At the end of the day that’s all you can do.”

Also:

Provider 1: “If I know a patient is drinking a lot and it’s a problem, it’s going to interfere with what medication their going to take. I’m not going to prescribe them medication that has very low barrier to resistance. So, I may choose a less wimpy regimen – something a bit stronger or stable because I would be worried about it if they keep missing doses they eventually build up resistance. I want something a bit more aggressive in terms of regimen.”

Some providers (n = 3; NP, MD) reported concerns discontinuing ART altogether because of the contraindication with alcohol:

Provider 10: “As [your] healthcare provider, responsibly, I can’t continue to provide you with these medications until we evaluate you for liver failure. The client perks up to that and is like ‘So, I really have a problem?’ [Provider says], ‘Yeah, whether it’s drinking or not, you have a liver problem.’ It’s good to have something wrong because they can see it. It’s tangible.”

Some providers (n = 3, CA, NP, MD) also felt that, aside from HIV-related outcomes, alcohol consumption had a detrimental effect on behavioral/mental health that often affected the level of care that could be provided in the clinic setting:

Provider 12: “If the provider feels that it is not an appropriate time to see the patient because they may not understand the instructions they are getting [due to alcohol use], we’ll just reschedule the appointment. We don’t want to service a client that is under substance abuse, or not acknowledging services they are receiving”

Also:

Provider 9: “I’ll just say ‘Okay, this appointment is over. I will not discuss your healthcare with you while you are under the influence of drugs. If you want your information, come back sober”.

Related, a clinical administrator expressed concern that the primary provider does not engage patients well enough to address the underlying issue. This provider also reported perceptions of stigmatization of patients with unhealthy alcohol use.

Provider 12: “I think sometimes, this is my opinion that maybe the provider gives up too fast. You know, we know alcohol abuse is a disease and I think that sometimes here maybe the provider feels, [well, if you’re not taking care of your HIV, how are you going to take care of your other stuff?]. – When I say give up, I mean maybe they are like [I’m not going to see the patient because they are drinking] – you know? Or they’ve missed too many appointments, because they are probably home drinking.”

As mentioned above, most providers did perceive a link between alcohol consumption and poor health outcomes, related to medical non-adherence, viral load, and behavioral issues. However, there was a subset of providers (n = 4, PA, MD) that reported no such link among their patients who engage in light to moderate alcohol use:

Provider 8: “Moderate use, I don’t really see any kind of correlation, because they take their meds and you know, they might have a beer at night and that’s it.”

(4) *Providers reported inconsistent recommendations regarding alcohol use* When asked to describe the recommendations given to their patients regarding level of alcohol use, some providers (n = 5; MA, MD) reported abstinence as their recommendation, citing ART adherence and organ failure to be the main reason for this recommendation:

Provider 3: “I never recommend a safe level of alcohol use. If they have hepatitis C or hepatitis B, I ride them hard to give up alcohol and to avoid Tylenol and stuff. Now one of the things you have to realize is alcohol is one of the number one causes of fatty liver disease… steatohepatitis. Most of my patients end up having that. They’re obese or they were on old-time HIV medications that also caused fat to deposit in the liver. I’m very clear – zero alcohol.”

More providers (n = 8; PA, NP, MD) felt that, if alcohol is to be consumed, light to moderate alcohol consumption was a reasonable recommendation to their patients. Providers with this recommendation seemed to provide patients with information regarding how much low to moderate drinking is and what level of drinking they recommend their patients not exceed, if they are to drink:

Provider 3: “I tell them, ‘I really don’t want to see you using more than 2 grams of alcohol. If you’re going to drink, I’d really prefer that you not drink every day, and if you do drink, I’d prefer you to limit it to two drinks, either a couple of ounces of liquor or the 12 oz beers. That 32 oz beer is more than one beer. I do encourage them to think about it. Sometimes it’s a slippery slope that can lead to more drinking. That’s what we talk about”.

Other providers feel moderate drinking is acceptable, but only if HIV viral load and other related comorbidities are well managed:

Provider 10: “That one or two glasses of wine, or one or two beers, or one or two cocktails – I’m okay with that as long as your liver enzymes are normal and you’re doing well. But, if your numbers start to change, if I see a problem, I’m going to let you know… I’m not against [drinking]. I am not an anti-drinker, but I do encourage that they consider the amount… and don’t miss your medicine. Take your medicine anyway.”

Some providers (n = 5; PA, NP, MD) reported that, if their patients engage in unhealthy alcohol use, they try to plan for alcohol reduction as opposed to complete abstinence:

Provider 8: “So, if they’re heavy users you never try to tell them to quit – right – you try to bargain. ‘Well alright – if you have six today, would you mind having maybe only four tomorrow, or four and a half? Like throw out the last half of that last can.’ Because, if you tell them anything about changing it doubles instead – so you work things out. You have to bargain with them”

A couple of providers (n = 2; NP, MD) focus on referrals for those patients that engage in unhealthy alcohol use, as opposed to making quit or reduction plans:

Provider 7: “Some of them, they will relapse very easily, and it is hard, that’s why I refer my patients to somebody else. Because, I try. All of the times I’ve tried to help somebody quit, I cannot do it. I think it’s more intense in-patient than for them to just be told once every three months that they should just stop, or they should decrease the amount of alcohol.”

Some providers (n = 4; MA, NP, MD) reported trying to educate the patient on the effects that alcohol use may have on their health and life, in lieu of an alcohol consumption recommendation:

Provider 3: “What I try to say [is], ‘Even if your alcohol use isn’t affecting your HIV care, it seems to be affecting your life. You have fights with your partners or you’ve gotten a DUI [driving under the influence]. These are some serious consequences from your drinking that to me, suggests that you might have a problem. Do you think you have a problem?’ We talk about what other things are going through the liver or we talk about the HIV medications – all of them require your liver to be healthy, for the most part. If you’re on any regimen, it’s going to need a healthy liver.”

(5) *Providers have limited resources for patients with unhealthy alcohol use* Providers were asked to describe what resources or treatment options they currently have in order to address unhealthy alcohol use. Some providers were able to identify one or two local alcohol programs (n = 5; CA, NP, MD; Alcoholics Anonymous [AA], Drug Abuse Comprehensive Coordinating Office [DACCO], South Florida AIDS Network) or mental health professions (n = 4; MA, NP, MD) that were available either as a referral or within the same healthcare unit to help patients with unhealthy alcohol use:

Provider 3: “I usually print out a list of local places that they can have access to, Alcoholics Anonymous meetings they need – AA. I give everybody the AA part because I figure it doesn’t matter what you’re doing, it might help.”

Also:

Provider 13: “We have psychologists that come here in the afternoon. We have social workers that work with the patient. The patient can be seen the same day, or sometimes they just come and even when they don’t have an appointment – we try to get an appointment either with the social worker or the psychologist.”

Several providers (n = 6; PA, MD) reported significant barriers to helping patients with unhealthy alcohol use, ranging from lack of resources to lack of patient motivation:

Provider 3: “I don’t have a lot of resources for referral. There really are limited resources in some of these rural areas for folks for even AA meetings when they don’t have transportation.”

Also:

Provider 2: “It is very difficult to get [patients] to go to a DACCO or another session. Most of the time, people don’t want it. They, I think, are treating their concomitant depression or mania – and say ‘No. I like alcohol as my drug. I’m not going to change.’”

(6) *Providers have low confidence in their ability to help patients reduce alcohol use* Several providers (n = 5; CA, NP, MD) mentioned low confidence in their training or little experience helping patients with unhealthy alcohol use. One provider mentioned lack of clinical training as a barrier to providing interventional support for their patients who engage in unhealthy alcohol use:

Provider 3: “I probably am not very effective in alcohol counseling. The reality is that it is not something that I was trained in… an advocate and maybe somebody that actually knows how to empower [patients] better than I do… maybe I don’t know the right way to encourage people.”

One provider mentioned lack of personal experience as a barrier:

Provider 9: “See, I’m not the expert in substance abuse. I’ve never used drugs, I’m not an alcohol drinker – a little bit of wine will do me – I never smoked and I don’t have the background of the people that use these. I don’t know what possessed them to start drinking. So, I’m totally clueless… I would find it better to have somebody that I can [send] them to and have them deal with it – somebody who really understands the whole dynamics. Because, they do a better job. I can tell them ‘don’t do it, stop your alcohol use,’ but what tools do I have to give them to do this? I know nothing.”

## Discussion

Results from our study suggest salient differences in provider perceptions and systemic approaches to managing unhealthy alcohol use among PLWH. This included a lack of uniformity in policies and procedures among HIV primary care providers for assessing unhealthy alcohol use. For example, some providers reported assessing alcohol use at every visit, at the first appointment, or periodically. A similar theme has been demonstrated in general primary care-in which alcohol assessment was not standardized and/or not consistent in assessment period across providers [33, 34, 37]. Our findings are also consistent with results from a survey of HIV care providers, finding that one-third reported usually asking about alcohol use frequency and quantity [31]. Related, many providers expressed concerns regarding the accuracy of self-report, which may be influenced by patient–provider rapport. Consistent with this finding, previous literature has found the patient–provider relationship as an important determinant of patients’ comfort in disclosing behaviors, such as unhealthy alcohol use, in primary care settings [33, 37].

While most providers reported that alcohol use deleteriously affects medication adherence, in line with studies showing a significant relationship between alcohol consumption and suboptimal ART adherence [2], providers reported conflicting alcohol use recommendations for patients. Nearly one-third endorsed an abstinence-only approach. In contrast, other providers were approving of light to moderate alcohol consumption, contingent on optimal ART adherence and no signs of adverse health issues, such as liver disease. Further, some providers expressed concern providing particular ART regimens or providing medication at all in the presence of unhealthy alcohol use and potential liver failure. Currently, there are inconsistent guidelines regarding safe levels of alcohol use [41]—which may be reflected in the inconsistent recommendations for alcohol use/non-use reported in the current study.

Many providers in our study also expressed doubt in their ability to effectively treat and refer those with unhealthy alcohol use to appropriate programs. This theme was further exemplified as a lack of formal education/training and resources necessary to intervene. This is consistent with qualitative research in general primary care [33–35, 37, 38] and quantitative research in HIV care [31] settings indicating that providers’ perception of experience and training in alcohol use treatment is a major barrier to brief intervention and treatment referrals. This may be compounded by a general lack of knowledge of medications/treatments for unhealthy alcohol use that are consistently effective with minimal side effects or contraindications [42], which was reported as a barrier in providing pharmacological treatment for unhealthy alcohol use in HIV care settings [31].

Our findings are consistent with previous work by Strauss et al. [43, 44], describing the state of alcohol reduction support offered by HIV care providers in hospital-based HIV/AIDS centers utilizing Screening and Brief Intervention (SBI) procedures. Strauss et al. [44] found that barriers to implementation of SBI components included inaccurate patient self-report of alcohol use, inconsistent provider perspective on alcohol use, and provider specialization that discourages treatment of comorbid unhealthy alcohol use. Additionally, five providers in our study reported low confidence in their ability to effectively manage patient alcohol abuse. Along these lines, previous studies have shown that providers with limited confidence in their ability to provide assistance related to unhealthy alcohol use among PLWH were less likely to have a high level of role legitimacy (i.e., extent to which healthcare providers believe that treating substance abuse issues is their responsibility [45]). Ultimately, patients with unhealthy alcohol use are less likely to be satisfied with their care and overall patient–provider communication regarding their unhealthy alcohol use [46, 47].

Limited resources for unhealthy alcohol use interventions and conflicting provider reports about the effect of alcohol use on HIV-related health outcomes complicate the recommendations and treatment of this behavior [48]. Given the negative implications of alcohol use on ART adherence, in conjunction with self-doubt expressed by providers to effectively treat unhealthy alcohol use, these findings support calls for integrated substance use and mental health treatment providers in infectious disease and primary care clinics frequented by PLWH. Specifically, identification of unhealthy alcohol use and providing appropriate referrals for intervention may improve HIV-related outcomes, especially given findings from previous studies showing decreased alcohol consumption and improved ART adherence with components of brief-intervention [49, 50]. Additionally, HIV-care providers may benefit from training in motivational interviewing, a goal-oriented therapy approach for eliciting behavior change, as this style of point-of-care therapy has been associated with reduction in alcohol use quantity and frequency among PLWH [50, 51].

There are limitations to this study that readers should consider, as well as notable strengths. First, while we aimed to recruit until saturation of themes was achieved, we recognize that the current sample is small and further qualitative research should be conducted with larger samples of diverse providers. Second, self-selection bias among our provider participants is possible. The providers that agreed to participate may represent a subgroup of providers that are highly motivated to address alcohol use in their clinics. We made great effort to engage providers at multiple levels of care for depth and breadth of experience and opinion. Further, we utilized flyers, provider letters, and word of mouth to ensure that all qualifying providers would have an opportunity to participate. Third, patient perspectives on alcohol related issues and harm reduction services within HIV primary care were not examined. Therefore, further qualitative investigation is needed in order to understand the patient perspectives on alcohol use and preferred treatment strategies to reduce unhealthy alcohol use. Despite these limitations, notable and consistent themes emerged from a heterogeneous group of HIV-care providers across the state of Florida.

## Conclusions

With 43% of PLWH reporting unmet healthcare needs related to drug or alcohol use [52], support for addressing alcohol use within a primary care setting is of great importance and studies have demonstrated the usefulness of brief intervention to reduce unhealthy alcohol use [53]. As HIV-primary care providers continue to serve as both the specialist and the primary care point-of-contact for PLWH, it is recommended that clinicians assess all patients aged 18 years and older for unhealthy alcohol use, and to provide support to reduce unhealthy alcohol consumption [54]. Use of standard definitions and diagnostic measures will provide uniformity in the assessment of alcohol use [55], and will help to inform specific substance abuse treatments tailored to PLWH. Future practice steps should include educating patients on the adverse effects of consuming varying quantities of alcohol in relation to ART use, HIV disease progression, and the development of comorbid illnesses. Additionally, implementation of more readily accessible and effective alcohol treatment resources and intervention programs may strengthen provider self-efficacy in providing appropriate treatment and referrals for unhealthy alcohol use among PLWH.

## Data Availability

The data collected and analyzed for the current study are available from the senior author (Ennis) on reasonable request.

## Abbreviations

AA: alcoholics anonymous
AIDS: acquired immunodeficiency syndrome
ART: antiretroviral therapy
DACCO: Drug Abuse Comprehensive Coordinating Office
DUI: driving under the influence
HIV: human immunodeficiency virus
IV: intravenous
PLWH: persons living with HIV
SBI: screening and brief intervention
SHARC: Southern HIV and Alcohol Research Consortium

## References

[1] Saitz R (2005). Clinical practice. Unhealthy alcohol use. N Engl J Med.

[2] Hendershot CS, Stoner SA, Pantalone DW, Simoni JM (2009). Alcohol use and antiretroviral adherence: review and meta-analysis. J Acquir Immune Defic Syndr 1999.

[3] Chander G, Josephs J, Fleishman JA (2008). Alcohol use among HIV-infected persons in care: results of a multi-site survey. HIV Med.

[4] Kelso-Chichetto NE, Okafor CN, Harman JS, Canidate SS, Cook CL, Cook RL (2016). Complementary and alternative medicine use for HIV management in the state of Florida: medical monitoring project. J Altern Complement Med NY.

[5] Hartzler B, Dombrowski JC, Crane HM (2017). Prevalence and predictors of substance use disorders among HIV care enrollees in the United States. AIDS Behav.

[6] Vagenas P, Azar MM, Copenhaver MM, Springer SA, Molina PE, Altice FL (2015). The impact of alcohol use and related disorders on the HIV continuum of care: a systematic review: alcohol and the HIV continuum of care. Curr HIV/AIDS Rep.

[7] Williams EC, Hahn JA, Saitz R, Bryant K, Lira MC, Samet JH (2016). Alcohol use and human immunodeficiency virus (HIV) infection: current knowledge, implications, and future directions. Alcohol Clin Exp Res.

[8] Hahn JA, Samet JH (2010). Alcohol and HIV disease progression: weighing the evidence. Curr HIV/AIDS Rep.

[9] Azar MM, Springer SA, Meyer JP, Altice FL (2010). A systematic review of the impact of alcohol use disorders on HIV treatment outcomes, adherence to antiretroviral therapy and health care utilization. Drug Alcohol Depend.

[10] Wu ES, Metzger DS, Lynch KG, Douglas SD (2011). Association between alcohol use and HIV viral load. J Acquir Immune Defic Syndr 1999.

[11] Bagby GJ, Amedee AM, Siggins RW, Molina PE, Nelson S, Veazey RS (2015). Alcohol and HIV effects on the immune system. Alcohol Res Curr Rev.

[12] Baum MK, Rafie C, Lai S, Sales S, Page JB, Campa A (2010). Alcohol use accelerates HIV disease progression. AIDS Res Hum Retroviruses.

[13] Braithwaite RS, Bryant KJ (2010). Influence of alcohol consumption on adherence to and toxicity of antiretroviral therapy and survival. Alcohol Res Health J Natl Inst Alcohol Abuse Alcohol.

[14] Neuman MG, Schneider M, Nanau RM, Parry C (2012). Alcohol consumption, progression of disease and other comorbidities, and responses to antiretroviral medication in people living with HIV. AIDS Res Treat.

[15] Samet JH, Cheng DM, Libman H, Nunes DP, Alperen JK, Saitz R (2007). Alcohol consumption and HIV disease progression. J Acquir Immune Defic Syndr.

[16] Freiberg MS, McGinnis KA, Kraemer K (2010). The association between alcohol consumption and prevalent cardiovascular diseases among HIV-infected and HIV-uninfected men. J Acquir Immune Defic Syndr 1999.

[17] Freiberg MS, Chang C-CH, Kuller LH (2013). HIV infection and the risk of acute myocardial infarction. JAMA Intern Med.

[18] Kelso NE, Sheps DS, Cook RL (2015). The association between alcohol use and cardiovascular disease among people living with HIV: a systematic review. Am J Drug Alcohol Abuse..

[19] McGinnis KA, Fultz SL, Skanderson M, Conigliaro J, Bryant K, Justice AC (2006). Hepatocellular carcinoma and non-Hodgkin’s lymphoma: the roles of HIV, hepatitis C infection, and alcohol abuse. J Clin Oncol Off J Am Soc Clin Oncol.

[20] Anand P, Springer SA, Copenhaver MM, Altice FL (2010). Neurocognitive impairment and HIV risk factors: a reciprocal relationship. AIDS Behav.

[21] Persidsky Y, Ho W, Ramirez SH (2011). HIV-1 infection and alcohol abuse: neurocognitive impairment, mechanisms of neurodegeneration and therapeutic interventions. Brain Behav Immun.

[22] Thaler NS, Sayegh P, Kim MS, Castellon SA, Hinkin CH (2015). Interactive effects of neurocognitive impairment and substance use on antiretroviral non-adherence in HIV disease. Arch Clin Neuropsychol Off J Natl Acad Neuropsychol.

[23] Braithwaite RS, Conigliaro J, Roberts MS (2007). Estimating the impact of alcohol consumption on survival for HIV+ individuals. AIDS Care.

[24] Justice AC, McGinnis KA, Tate JP (2016). Risk of mortality and physiologic injury evident with lower alcohol exposure among HIV infected compared with uninfected men. Drug Alcohol Depend.

[25] Neblett RC, Hutton HE, Lau B, McCaul ME, Moore RD, Chander G (2011). Alcohol consumption among HIV-infected women: impact on time to antiretroviral therapy and survival. J Womens Health 2002.

[26] Stockwell T, Zhao J, Panwar S, Roemer A, Naimi T, Chikritzhs T (2016). Do, “Moderate” drinkers have reduced mortality risk? A systematic review and meta-analysis of alcohol consumption and all-cause mortality. J Stud Alcohol Drugs.

[27] Wandeler G, Kraus D, Fehr J (2016). The J-curve in HIV: low and moderate alcohol intake predicts mortality but not the occurrence of major cardiovascular events. J Acquir Immune Defic Syndr 1999.

[28] Cheng QJ, Engelage EM, Grogan TR, Currier JS, Hoffman RM (2014). Who provides primary care?.

[29] Montague BT, Kahler CW, Colby SM (2015). Attitudes and training needs of New England HIV care and addiction treatment providers: opportunities for better integration of HIV and alcohol treatment services. Addict Disord Their Treat.

[30] Conigliaro J, Gordon AJ, McGinnis KA, Rabeneck L, Justice AC (2003). Veterans aging Cohort 3-site study. How harmful is hazardous alcohol use and abuse in HIV infection: Do health care providers know who is at risk?. J Acquir Immune Defic Syndr.

[31] Chander G, Monroe AK, Crane HM (2016). HIV primary care providers–screening, knowledge, attitudes and behaviors related to alcohol interventions. Drug Alcohol Depend.

[32] Williams EC, Lapham GT, Shortreed SM (2017). Among patients with unhealthy alcohol use, those with HIV are less likely than those without to receive evidence-based alcohol-related care: a national VA study. Drug Alcohol Depend.

[33] McNeely J, Kumar PC, Rieckmann T (2018). Barriers and facilitators affecting the implementation of substance use screening in primary care clinics: a qualitative study of patients, providers, and staff. Addict Sci Clin Pract.

[34] Williams EC, Achtmeyer CE, Young JP (2016). Local implementation of alcohol screening and brief intervention at five veterans health administration primary care clinics: perspectives of clinical and administrative staff. J Subst Abuse Treat.

[35] Johnson M, Jackson R, Guillaume L, Meier P, Goyder E (2011). Barriers and facilitators to implementing screening and brief intervention for alcohol misuse: a systematic review of qualitative evidence. J Public Health Oxf Engl.

[36] Young JP, Achtmeyer CE, Bensley KM, Hawkins EJ, Williams EC (2018). Differences in perceptions of and practices regarding treatment of alcohol use disorders among VA primary care providers in urban and rural clinics. J Rural Health Off J Am Rural Health Assoc Natl Rural Health Care Assoc.

[37] Williams EC, Achtmeyer CE, Thomas RM (2015). Factors underlying quality problems with alcohol screening prompted by a clinical reminder in primary care: a multi-site qualitative study. J Gen Intern Med.

[38] Williams EC, Achtmeyer CE, Young JP (2018). Barriers to and facilitators of alcohol use disorder pharmacotherapy in primary care: a qualitative study in five VA clinics. J Gen Intern Med.

[39] Edelman EJ, Hansen NB, Cutter CJ (2016). Implementation of integrated stepped care for unhealthy alcohol use in HIV clinics. Addict Sci Clin Pract.

[40] Saunders B, Sim J, Kingstone T (2018). Saturation in qualitative research: exploring its conceptualization and operationalization. Qual Quant.

[41] Kalinowski A, Humphreys K (2016). Governmental standard drink definitions and low-risk alcohol consumption guidelines in 37 countries. Addict Abingdon Engl.

[42] Substance Abuse and Mental Health Services Administration and National Institute on Alcohol Abuse and Alcoholism. Medication for the treatment of alcohol use disorder: a brief guide. HHS Publication No. (SMA) 15-4907. Rockville, MD: Substance Abuse and Mental Health Services Administration, 2015.

[43] Strauss SM, Tiburcio NJ, Munoz-Plaza C (2009). HIV care providers’ implementation of routine alcohol reduction support for their patients. AIDS Patient Care STDs.

[44] Strauss SM, Munoz-Plaza CE, Tiburcio NJ, Gwadz M (2012). Barriers and facilitators in implementing “prevention for positives” alcohol-reduction support: the perspectives of directors and providers in hospital-based HIV care centers. J Assoc Nurses AIDS Care JANAC.

[45] Strauss SM, Munoz-Plaza C, Tiburcio NJ (2009). HIV care providers’ role legitimacy as supporters of their patients’ alcohol reduction. Open Infect Dis J.

[46] Korthuis PT, Saha S, Chander G (2011). Substance use and the quality of patient–provider communication in HIV clinics. AIDS Behav.

[47] Ray MK, Beach MC, Nicolaidis C, Choi D, Saha S, Korthuis PT (2013). Patient and provider comfort discussing substance use. Fam Med.

[48] Armstrong ML, LaPlante AM, Altice FL, Copenhaver M, Molina PE (2015). Advancing behavioral HIV prevention: adapting an evidence-based intervention for people living with HIV and alcohol use disorders. AIDS Res Treat.

[49] Holstad MM, DiIorio C, Kelley ME, Resnicow K, Sharma S (2011). Group motivational interviewing to promote adherence to antiretroviral medications and risk reduction behaviors in HIV infected women. AIDS Behav.

[50] Nyamathi A, Shoptaw S, Cohen A (2010). Effect of motivational interviewing on reduction of alcohol use. Drug Alcohol Depend.

[51] Brown JL, DeMartini KS, Sales JM, Swartzendruber AL, DiClemente RJ (2013). Interventions to reduce alcohol use among HIV-infected individuals: a review and critique of the literature. Curr HIV/AIDS Rep.

[52] Krause DD, May WL, Butler KR (2013). Determining unmet, adequately met, and overly met needs for health care and services for persons living with HIV/AIDS in Mississippi. AIDS Care.

[53] Jonas DE, Garbutt JC, Amick HR (2012). Behavioral counseling after screening for alcohol misuse in primary care: a systematic review and meta-analysis for the U.S. Preventive Services Task Force. Ann Intern Med.

[54] Final Update Summary: Alcohol misuse: screening and behavioral counseling interventions in primary care—US Preventive Services Task Force. https://www.uspreventiveservicestaskforce.org/Page/Document/UpdateSummaryFinal/alcohol-misuse-screening-and-behavioral-counseling-interventions-in-primary-care. Accessed 3 Oct 2016.

[55] Saitz R, Saxon A, Hermann R. Screening for unhealthy use of alcohol and other drugs in primary care. 2016. https://www.uptodate.com/contents/screening-for-unhealthy-use-of-alcohol-and-other-drugs-in-primary-care/print. Accessed 3 Oct 2016.

